# Inhibition of Calcium Oxalate Formation and Antioxidant Activity of Carboxymethylated *Poria cocos* Polysaccharides

**DOI:** 10.1155/2021/6653593

**Published:** 2021-03-01

**Authors:** Chuang-Ye Li, Li Liu, Yao-Wang Zhao, Jia-Yun Chen, Xin-Yuan Sun, Jian-Ming Ouyang

**Affiliations:** ^1^Department of Urology, Hunan Children's Hospital, Changsha 410007, China; ^2^Institute of Biomineralization and Lithiasis Research, Jinan University, Guangzhou 510632, China

## Abstract

Three carboxymethylated *Poria cocos* polysaccharides (PCP-C1, PCP-C2, and PCP-C3) with -COOH contents of 6.13%, 10.24%, and 16.22%, respectively, were obtained by carboxymethylation of the original polysaccharide (PCP-C0), which has a molecular weight of 4 kDa and a carboxyl (-COOH) content of 2.54%. The structure of the PCP-Cs was characterized by FT-IR, ^1^H NMR, and ^13^C NMR spectra. The four PCP-Cs exhibited antioxidant activity, and their ability to scavenge radicals (hydroxyl and DPPH) and chelate ferrous ions was positively correlated with the degree of carboxymethylation. As the content of -COOH groups in the PCP-Cs increases, their ability to regulate the growth of calcium oxalate (CaOx) crystals was enhanced, thus inhibiting the growth of calcium oxalate monohydrate (COM) crystals and inducing the formation of more calcium oxalate dihydrate (COD) crystals. The formed CaOx crystal was more round and blunt, the absolute value of the Zeta potential on the crystal surface increased, and the aggregation between crystals was inhibited. Thermogravimetric analysis curves showed that the proportions of PCP-C0, PCP-C1, PCP-C2, and PCP-C3 incorporated into the crystal were 20.52%, 15.60%, 10.65%, and 9.78%, respectively, in the presence of 0.4 g/L PCP-Cs. PCP-C protection resisted oxidative damages of human kidney proximal tubular epithelial cells (HK-2) caused by oxalate, resulting in increased cell viability and superoxide dismutase activity and decreased reactive oxygen species levels, malondialdehyde content, and 8-hydroxy-deoxyguanosine expression. Hence, PCP-Cs, especially PCP-C3, can inhibit the formation of CaOx crystals and may have the potential to be an alternative antistone drug.

## 1. Introduction

Kidney stones are a very common disease and are believed to be the result of the interaction between the genetic and environmental factors. About 80% of kidney stones are calcium oxalate (CaOx) stones, and the two main forms are calcium oxalate monohydrate (COM) and calcium oxalate dihydrate (COD) [1]. COM is more likely to damage renal epithelial cells [2]; the cell damage will promote the nucleation, growth, aggregation, and adhesion of crystals on its surface [3], thereby increasing the formation risk of kidney stones.

Polysaccharides are widely found in plants, animals, and microorganisms. Natural polysaccharides possess antioxidant, antidiabetic, antitumor, and other biological activities. The biological activity of polysaccharides is usually related to their molecular weight and chain structure, especially the number of active groups [4, 5].

Biomacromolecules (such as polysaccharides) rich in acidic polyanionic groups can be used as effective inhibitors of CaOx calculus formation. Zhang et al. [6] found that 0.5 g/L *Sargassum* polysaccharide can inhibit the growth and aggregation of COM crystals and induce the formation of spherical COD crystals; the inhibition rates of crystal nucleation and aggregation are 69.2% and 76.8%, respectively.

The biological activity of polysaccharides is closely related to the content of acidic groups [7–10]. Xu et al. [9] extracted two polysaccharides from *E*. *acuminatum*; the neutral polysaccharide EAP-1N has a uronic acid content of 0.32%, and the acid polysaccharide EAP-2A has a uronic acid content of 9.46%. EAP-2A has a stronger ability to scavenge free radicals, enhance the activity of antioxidant enzymes (including superoxide dismutase (SOD), catalase (CAT), and glutathione peroxidase (GSH-Px)), and inhibit lipid peroxidation. Huang et al. [10] compared the effects of five plant polysaccharides (PPSs) with -COOH contents of 6.3%, 8.9%, 9.1%, 13.6%, and 16.1%, respectively, on the growth of CaOx crystals; the ability of PPSs to inhibit CaOx growth and aggregation and induce COD formation was positively correlated with the percentage of -COOH groups in the polysaccharide.

Chemically modified polysaccharides show stronger biological activity than natural polysaccharides [11–15]. Wang et al. [11] modified *Poria cocos* polysaccharide by carboxymethylation and reported that the polysaccharide with the highest carboxymethylation degree had the highest chelating ability to ferrous ions and the highest scavenging ability to hydroxyl radicals. Wang et al. [13] prepared carboxymethylated polysaccharide (CATP) from *Tremella* polysaccharide (ATP) and found that CATP had significantly higher antioxidant activity and improved water solubility than ATP. Li et al. [15] treated hypercholesterolemic rats with a high dose of *Morchella angusticeps* Peck polysaccharide (PMEP) and its carboxymethylated polysaccharide (CPMEP) and reported that the serum total cholesterol levels of rats were 1.54 and 1.29 mmol/L, respectively; hence, CPMEP has stronger cholesterol-lowering activity and can upregulate the protein expression of CYP7A1 and LDL-R in rat livers, downregulate the expression of HMG-CoA, and improve its cholesterol-lowering ability.

*P*. *cocos* polysaccharide (PCP) is one of the main components of *P*. *cocos* [16] and can be used to treat chronic gastritis, nephropathy, dizziness, and vomiting [17]. PCP is mainly composed of four monosaccharides, namely, D-glucose, D-mannose, D-fucose, and D-xylose [18, 19]. Wang et al. [20] found that PCP extracted by an ultrasonic wave has the ability to reduce power and scavenge hydroxyl and DPPH radicals. Wu et al. [21] showed that PCP can inhibit acetaminophen-induced liver injury in mice and reduce the levels of the serological liver enzyme (ALT), lactate dehydrogenase (LD), and inflammatory cytokines (TNF-*α*, IL-6).

In this study, three carboxymethylated polysaccharides (PCP-C1, PCP-C2, and PCP-C3) with -COOH contents of 6.13%, 10.24%, and 16.22%, respectively, were obtained by carboxymethylation of *Poria* polysaccharide (PCP-C0) with initial -COOH content of 2.54%. Inhibitory effects on the formation of calcium oxalate crystals were studied in vitro to obtain potential anticalculus polysaccharide drugs.

## 2. Experimental Methods

### 2.1. Materials and Apparatus

#### 2.1.1. Materials

*Poria cocos* polysaccharide (PCP-C0) was produced by Shaanxi Ciyuan Biological Company, with polysaccharide content ≥ 95%; the protein present in polysaccharide was removed by the Sevag method; after treating the polysaccharide with the chloroform-n-butanol mixed solution, the free protein is denatured into insoluble substances and the purpose of separation is achieved [22]. Phosphate-buffered solution (PBS), phenazine, 1,1-diphenyl-2-picrylhydrazyl (DPPH), phenanthroline, and other conventional reagents were of analytical grade and were purchased from Guangzhou Chemical Reagent Company (Guangzhou, China). The Cell Counting Kit-8 (CCK-8) was purchased from Dojindo Laboratory (Kumamoto, Japan). 2′,7′-Dichlorodihydrofluorescein diacetate (DCFH-DA) was purchased from Shanghai Beyotime Bio-Tech Co., Ltd. (Shanghai, China). The malonaldehyde (MDA) kit and superoxide dismutase (SOD) kit were purchased from Jiancheng Institute of Biotechnology (Nanjing, China). The Human 8-hydroxy-deoxyguanosine (8-OHdG) ELISA Kit was purchased by Boshen Biotechnology (Nanjing, China). The experimental water is double-distilled water.

#### 2.1.2. Apparatus

The apparatus used in the study included the following: Fourier transform infrared spectrometer (FT-IR, Nicolet, USA); nuclear magnetic resonance (Varian Bruker-500 MHz, Bruker, Germany); ultrasonic apparatus (aligned with JP-100S); Ubbelohde capillary viscometer (0.4–0.5, Qihang Glass Instrument Factory, Shanghai, China); thermogravimetric analyzer (TGA/DSC 3+, Mettler Toledo, USA); D/max 2400-X-ray powder diffractometer (Rigaku, Japan); field emission scanning electron microscope (ULTRA55, Zeiss, Germany); OPTIMA-2000DV inductively coupled plasma (ICP) (ICP-AES, Optima 2000DV, PerkinElmer, CT, USA); Zetasizer 300HS nanoparticle size-Zeta potential analyzer (Malvern, UK); and Enzyme Mark Instrument (Safire^2^, Tecan, Switzerland).

### 2.2. Carboxymethylation and Characterization of *Poria cocos* Polysaccharides (PCP-C0)

#### 2.2.1. Carboxymethylation of PCP-C0 [23–25]

300 mg PCP-C0 was suspended in 15 mL of isopropanol, stirred at room temperature for 15 min, slowly added 10 mL of 30% NaOH solution, stirred at 50°C water bath for 1 h, then added 2.63 g of monochloroacetic acid, and then heated at 60°C for 2 h. After cooling to room temperature, adjust to neutrality with 0.5 mol/L HCl, dialyze with distilled water (Mw retention 3000 Da) for 3 days, and concentrate and freeze-dry to obtain carboxymethylated polysaccharides (PCP-Cs) with different degrees of substitution.

#### 2.2.2. Determination of -COOH Content in PCP-Cs [26]

-COOH content of PCP-Cs was determined by the method of conductometric titration. The conductivity titration curve was plotted using the conductivity value as the *Y*-axis and the used NaOH volume as the *X*-axis. From the conductivity titration curve, the volume of NaOH consumed by polysaccharide-COOH is obtained, and the percentage of -COOH is calculated. Each experiment was repeated three times.

#### 2.2.3. FT-IR Characterization of PCP-Cs

FT-IR spectra of polysaccharides were determined using films prepared by 2.0 mg of the dry PCP-C sample and 200 mg KBr in the wavenumber range of 4000-400 cm^−1^.

#### 2.2.4. ^1^H and ^13^C NMR Characterization of PCP-Cs

Approximately 20 mg of the dry PCP-C sample was dissolved in 0.5 mL of deuterated water (D_2_O) in an NMR tube. After being completely dissolved, the polysaccharide sample is put into a magnetic field of a nuclear magnetic resonance spectrometer for detection.

### 2.3. Antioxidant Activity Detection of PCP-Cs

#### 2.3.1. Hydroxyl Radical (^·^OH) Scavenging Capacity

The ^·^OH scavenging ability of polysaccharides in vitro was detected by the H_2_O_2_/Fe system method [27]. Add 1 mL 2.5 mmol/L FeSO_4_ solution and 1 mL 2.5 mmol/L phenanthroline solution to the test tube, then add PBS (20 mmol/L, 1 mL, pH = 7.4) and 1 mL 20 mmol/L H_2_O_2_ in sequence, and finally take 1 mL of different concentrations of polysaccharide samples (0.15, 0.5, 0.8, 1.0, 2.0, and 3.0 g/L) into test tubes. After mixing well, incubate at 37°C for 90 min and use a UV spectrophotometer to measure the absorbance at 536 nm; the average absorbance is *A*_3_. Each experiment was repeated three times. The absorbance when hydrogen peroxide (H_2_O_2_) and polysaccharide solution were replaced with distilled water was *A*_2_. The absorbance when polysaccharide solution was replaced with distilled water was *A*_1_. The ascorbic acid (VC) was used as the positive control group. The ability to scavenge hydroxyl radicals was calculated using the following equation:

(1) $$\begin{array}{c} \text{Scavenging effect }\left(\%\right)=\frac{\left(A_{3}-A_{1}\right)}{\left(A_{2}-A_{1}\right)}\times 100\%.. \end{array}$$

#### 2.3.2. DPPH Radical Scavenging Capacity [28, 29]

0.4 mmol/L DPPH was prepared by anhydrous ethanol, and 3 mL PCP-C (0.15-3 g/L) polysaccharide was mixed with DPPH (0.4 mmol/L, 1 mL). The mixture was incubated in the dark at 25°C for 30 min. The absorbance is detected at 517 nm to reflect the DPPH radical scavenging ability of polysaccharides. Each experiment was repeated three times.

(2) $$\begin{array}{c} \text{Scavenging effect }\left(\%\right)=\left[1-\frac{\left(A_{2}-A_{1}\right)}{A_{0}}\right]\times 100\%,. \end{array}$$

where *A*_2_ was the absorbance of 3 mL sample solution+1 mL DPPH reagent, *A*_1_ was the absorbance of 3 mL sample solution+1 mL anhydrous ethanol, and *A*_0_ was the absorbance of 1 mL DPPH solution+3 mL water.

#### 2.3.3. Ferrous Ion Chelating Capacity [30]

1 mL of PCP-Cs (0.15-3.0 g/L) was mixed with 2.25 mL distilled water and 0.05 mL 2.0 mmol/L ferrous chloride solution, respectively, and the reaction lasts for 30 s. Next, the solutions were mixed with 0.2 mL 5.0 mmol/L phenazine and reacted at room temperature for 10 min, and the absorbance of the mixture was measured at 562 nm. The experiment used water instead of polysaccharide solution and ferrous chloride solution as the blank group and control group. EDTA-2Na was used as the positive control group. Lower absorbance indicated a stronger chelating capacity for ferrous ions. Each experiment was repeated three times.

(3) $$\begin{array}{c} \text{Polysaccharide chelating ability }\left(\%\right)=\frac{\left[A_{0}-\left(A_{1}-A_{2}\right)\right]}{A_{0}}\times 100,. \end{array}$$

where *A*_0_ is the absorbance of the blank group, *A*_1_ is the absorbance of the sample group, and *A*_2_ is the absorbance of the control group.

### 2.4. Regulation of CaOx Crystal Growth by PCP-Cs [31]

#### 2.4.1. CaOx Crystal Synthesis

CaOx metastable solution was carried out in a 50 mL volumetric flask by adding 3.0 mL of 10 mmol/L CaCl_2_ and 1 mL of 0.50 mol/L NaCl; then, add a certain amount of PCP-C solution to make the final concentrations of polysaccharide: 0.05, 0.1, 0.2, 0.4, and 0.8 g/L, add distilled water to about 45 mL, then add 3.0 mL of 10 mmol/L Na_2_Ox, and finally dilute volume to scale with distilled water. The solution thus obtained is *c*(Ca^2+^) = *c*(Ox^2−^) = 0.60 mmol/L and *c*(NaCl) = 10 mmol/L. The crystal was filtered by a 0.22 *μ*m microporous membrane. The above CaOx solution was poured into a 50 mL beaker for crystallization, and a clean glass slide was placed at the bottom of the beaker. In order to prevent supersaturation of the system due to volatilization of solvent water from driving crystal formation, crystal growth was carried out at static conditions. After the crystal grows for 3 d, the substrate was taken out and dried in a dryer. The concentration of soluble Ca^2+^ ions in the supernatant was measured through inductively coupled plasma (ICP) emission spectrometry.

The above synthesized CaOx crystals were characterized by Fourier infrared (FT-IR), field emission scanning electron microscope (SEM), X-ray diffraction (XRD), and Zeta potentiometer.

#### 2.4.2. Characterization of SEM

The samples were treated with gold spray and observed under a field emission scanning electron microscope for morphology analysis.

#### 2.4.3. Characterization of FT-IR

The dried CaOx sample (2.0 mg) was mixed with KBr (200 mg), followed by grinding with agate mortar, tableting, and scanning with an infrared spectrometer in the wavenumber range of 4000-400 cm^−1^.

#### 2.4.4. Characterization of XRD

The synthesized crystals were analyzed in an X-ray diffractometer under the test conditions of CuK*α* ray, graphite monochromator, 30 kV, 25 mA, scanning range of 5-60°, scanning speed of 8°/min, and step width of 0.02°/s for qualitative and quantitative analyses.

The relative percentage contents of COM and COD in the CaOx precipitates were calculated through the *K* value method [31] and the relative percentage contents of COD:

(4) $$\begin{array}{c} \text{COD}\%=\frac{I_{\text{COD}}}{I_{\text{COD}}+I_{\text{COM}}}, \end{array}$$

where *I*_COM_ and *I*_COD_ are the intensities of the spacing $\left(\bar{1}01\right)$ of COM and (200) of COD crystals.

#### 2.4.5. Zeta Potential Determination of the Crystal Surface

CaOx crystals (1 mg) were dispersed in 3.0 mL of double-distilled water. After ultrasonication for 10 min, the Zeta potential was detected with a Zetasizer Nano ZS90 apparatus at 25°C.

#### 2.4.6. TGA for Determinating Polysaccharide Content in Crystals

According to Reference [26], the thermogravimetric analysis curves of CaOx crystals formed in the presence or absence of polysaccharides were measured by a Mettler Toledo thermal analyzer under a nitrogen atmosphere from 25°C to 900°C at a heating rate of 10°C/min. The polysaccharide content in the crystals was calculated from the thermal curves.

### 2.5. Cell Experiments

#### 2.5.1. Cell Culture and Cytotoxicity Detection of PCP-Cs

HK-2 cells were cultured in DMEM-F12 containing 10% fetal bovine serum [32]. Cell suspension (density: 1 × 10^5^ cells/mL) was inoculated into 96-well plates to make cells assemble into a monolayer; then, PCP-C0, PCP-C1, PCP-C2, and PCP-C3 with final concentrations of 20, 40, 60, 80, and 100 *μ*g/mL were added to cells, respectively. After 24 h culture, the cell viability was measured by CCK-8. Absorbance (*A*) was measured at 450 nm by an enzyme reader according to the kit measurement method, and the cell viability rate was calculated. Each experiment was repeated in five parallel wells.

#### 2.5.2. Cell Viability Detection [32]

After cells were confluent into the monolayer, the experiment was divided into 3 groups: (1) normal control group: only the serum-free culture medium was added; (2) damaged group: 2.8 mM of sodium oxalate was added, and cells were damaged for 3.5 h; and (3) protection group: PCP-C0, PCP-C1, PCP-C2, and PCP-C3 with final concentrations of 20, 40, 60, 80, and 100 *μ*g/mL were added to preprotect cells for 12 h, followed by 2.8 mM oxalate injury for 3.5 h. After incubating the above groups for 12 h, add 10 *μ*L of the CCK-8 reagent to each well and incubate at 37°C for 1.5 h. Absorbance (*A*) was measured by using the enzyme mark instrument at 450 nm. Each experiment was repeated in five parallel wells. Cell viability was determined using the equation below.

#### 2.5.3. Reactive Oxygen Species (ROS) Level Detection [32]

Cells were inoculated into 12-well culture plates at concentrations of 1.0 × 10^5^ cells/mL and 1 mL/well. The experimental model and grouping were the same as the CCK-8 experiment, and 100 *μ*g/mL of PCP-Cs was used to protect the cells. The cells were stained with 2′,7′-dichlorodihydrofluorescein diacetate (DCFH-DA) at 37°C for 30 min. The cells were washed with PBS three times to remove DCFH-DA that did not enter the cells. The fluorescence intensity was observed under a fluorescence microscope and also detected by a microplate reader. Each experiment was repeated in three parallel wells.

#### 2.5.4. Superoxide Dismutase (SOD) Activity Detection

SOD activity was assessed using a commercially available kit based on the autooxidation of hydroxylamine. The experimental model and grouping were the same as the CCK-8 experiment, and 100 *μ*g/mL of PCP-Cs was used to protect the cells. At the indicated time points, the treated cells were homogenized in 100 mmol/L Tris-HCl buffer and centrifuged at 10,000 rpm for 20 min, and then the SOD activity was determined using assay kits. The absorbance of the supernatant was then measured directly by a microplate reader at 560 nm with a reference wavelength of 600 nm.

#### 2.5.5. Malondialdehyde (MDA) Detection

For lipid peroxidation assay, we used a commercial kit to quantify the generation of MDA according to the manufacturer's protocol. The experimental model and grouping were the same as the CCK-8 experiment, and 100 *μ*g/mL of PCP-Cs was used to protect the cells. The cells were harvested by trypsinization, and cellular extracts were prepared by sonication in ice-cold buffer (50 mM Tris-HCl, pH = 7.5, 5 mM EDTA, and 1 mM DTT). After sonication, lysed cells were centrifuged at 10,000 rpm for 20 min to remove debris. The supernatant was subjected to the measurement of MDA levels by detecting the absorbance at 532 nm.

#### 2.5.6. 8-Hydroxy-Deoxyguanosine (8-OHdG) Detection

8-OHdG is a commonly used marker of oxidative DNA damage. The experimental model and grouping were the same as the CCK-8 experiment, and 100 *μ*g/mL of PCP-Cs was used to protect the cells. The concentration of 8-OHdG was measured using a commercial ELISA kit according to the manufacturer's instructions. The color change is measured spectrophotometrically at a wavelength of 450 nm.

### 2.6. Statistical Analysis

The normal distribution of experimental results was analyzed by the Shapiro–Wilk test. Data were assessed using a one-way ANOVA test, followed by Tukey's multiple comparison test for those following normal distribution. Experimental data were expressed by mean ± standard deviation$\left(\bar{x}\pm \text{SD}\right)$. The data were presented as individual values and assessed using the Kruskal–Wallis test, followed by Dunn's multiple comparison test when following a nonnormal distribution. If *P* < 0.05, there was a significant difference; if *P* < 0.01, the difference was extremely significant; if *P* > 0.05, there was no significant difference.

## 3. Results

### 3.1. Carboxymethylation and Characterization of *P*. *cocos* Polysaccharide (PCP-C0)

#### 3.1.1. Polysaccharide Carboxymethylation

**Figure 1:**
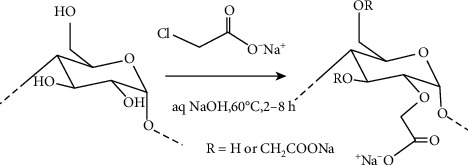
Carboxymethylation reaction of *Poria cocos* polysaccharide.

The carboxymethylation reaction belongs to bimolecular nucleophilic substitution (SN2) reactions [33]. First, the hydroxyl group of PCP-C0 was reacted with NaOH to form an alkoxide, and the carboxymethyl group was reacted with the alkoxide to form a carboxymethylated polysaccharide (PCP-C) (Figure 1).

#### 3.1.2. Detection of -COOH Content in PCP-Cs

**Figure 2:**
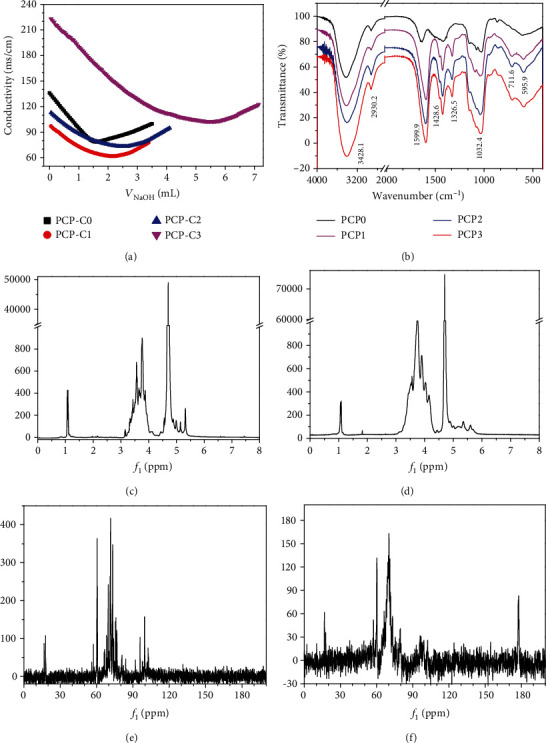
Characterization of PCP-Cs. (a) Titration curves for detecting -COOH content in four polysaccharides, *c*(PCP‐Cs) = 0.10 g/L and *c*(NaOH) = 5.24 mmol/L. (b) FT-IR spectra of four polysaccharides. (c, d) ^1^H NMR spectra of PCP-C0 and PCP-C1. (e, f) ^13^C NMR spectra of PCP-C0 and PCP-C1.

The -COOH content in each carboxymethylated polysaccharide (PCP-C) was determined by conductometric titration. The titration curves are shown in Figure 2(a). The -COOH contents of PCP-C0, PCP-C1, PCP-C2, and PCP-C3 were 2.54%, 6.13%, 10.24%, and 16.22%, respectively. After carboxymethylation modification, the molecular weight of the polysaccharide increased, but no obvious change in the molecular weight of the polysaccharide was detected [34].

#### 3.1.3. FT-IR Characterization of PCP-Cs

Figure 2(b) shows the FT-IR spectra of PCP-Cs with different degrees of carboxymethylation. The strong absorption band at 3428.1 cm^−1^ corresponds to the absorption peak of the stretching vibration of –OH in the polysaccharides. The absorption band at 2930.2 cm^−1^ corresponds to the stretching vibration of C-H. The peak at 1600 cm^−1^ corresponds to the stretching vibration of C-H-O and C-O bonds. The peak at 1428.6 cm^−1^ is the stretching vibration of the carboxyl group. Absorptions in the region of 1500-1000 cm^−1^ are attributed to epoxy vibration, (COH) side group stretching vibration, and C-O-C glycosidic bond vibration [35]. The peak at 1032.4 cm^−1^ corresponds to the stretching vibration of the pyranose ring of glucose residues [19].

After carboxymethylation of PCP-C0, a new absorption peak appeared at 1326.5 cm^−1^, which belongs to the absorption peak of carboxymethyl (-CH_2_COOH). According to the carboxymethylation reaction (Figure 1), the substitution of -CH_2_COOH occurred on the -OH group of polysaccharides and manifested as a new absorption peak near 1326.5 cm^−1^, thereby confirming the successful carboxymethylation [36].

#### 3.1.4. ^1^H NMR Characterization of PCP-Cs

In the ^1^H NMR spectrum of PCP-C0 (Figure 2(c)), *δ*5.14, 3.98, 3.67, 3.58, 3.81, and 3.66 ppm belong to the signal peaks of H-1 to H-6 of *α*-D-Gal; *δ*4.95, 3.59, 3.98, 3.89, 4.08, and 1.14 ppm belong to the signal peaks of H-1 to H-6 of *α*-(1-6)-L-Fuc (Table 1); *δ*4.95, 3.88, 3.75, 3.89, 4.08, and 1.14 ppm belong to the signal peaks of H-1 to H-6 of *α*-(1-3)-L-Fuc; *δ*4.71 ppm corresponds to the signal peak of H-1 of *β*-(1-3)-D-Glc; and *δ*4.59 ppm corresponds to the signal peak of H-1 of *β*-(1-3)-D-Gal [37, 38]. Compared with that of PCP-C0, the ^1^H NMR of the carboxymethylated polysaccharide PCP-C1 showed no new strong signal peaks (Figure 2(d)).

#### 3.1.5. ^13^C NMR Characterization of PCP-Cs

In the ^13^C NMR spectrum of PCP-C0 (Figure 2(e)), the strong signals at *δ*102.53, 74.12, 95.89, 69.58, 75.74, and 60.75 ppm belong to the C-1 to C-6 signal peaks of *β*-(1-3)-D-Glc (Table 2); *δ*103.7, 73.5, 86.9, 69.1, 77.0, and 61.6 ppm belong to the C-1 to C-6 signal peaks of *β*-(1-3)-D-Gal; *δ*73.6, 67.0, 70.5, and 61.3 ppm belong to the C-3 to C-6 signal peaks of *α*-D-Gal; *δ*98.2, 67.7, 70.5, 71.6, 67.3, and 15.8 ppm belong to the C-1 to C-6 signal peaks of *α*-(1-6)-L-Fuc; and *δ*98.2, 67.0, 69.7, 71.6, 67.3, and 15.8 ppm belong to the C-1 to C-6 signal peaks of *α*-(1-3)-L-Fuc [19, 37].

The main signals in the ^13^C NMR spectrum of PCP-C1 were 102.8 (C-1), 79.2 (C-3), 74.8 (C-5), 73.1 (C-2), 69.6 (C-4), 60.9 (C-6), and 177.8 ppm (C-7) (Figure 2(f)). The signals at 71.2 and 73.1 ppm are assigned to the methylene carbon atom by the carboxymethyl substituent. The signal increased at 70.6 ppm and decreased at 60.7 ppm, indicating the occurrence of carboxymethyl substitution at the C-6 position [36, 37]. For the ^13^C NMR of the carboxymethylated polysaccharide PCP-C1, a new signal peak appeared at 177.8 ppm, which belongs to the C=O bond of the carboxymethyl group (-CH_2_COOH); this finding can be used as evidence of the carboxymethylation reaction, consistent with previous reports [23, 36].

### 3.2. Antioxidant Capacity of PCP-Cs

#### 3.2.1. Scavenging Capacity of ^·^OH Radicals

**Figure 3:**
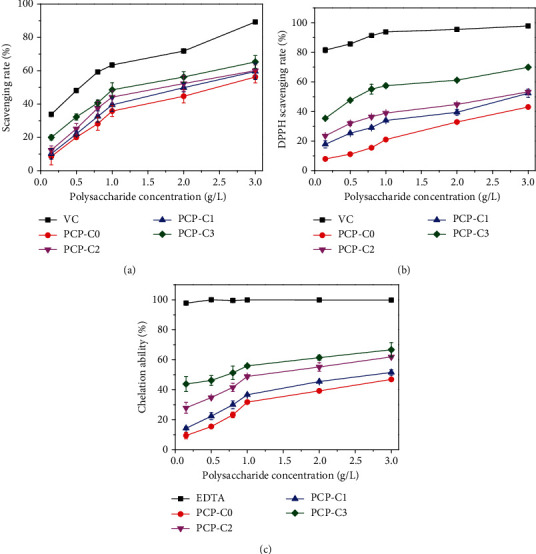
Antioxidant activity of different carboxymethylated *Poria cocos* polysaccharides (PCP-Cs). (a) PCP-C scavenging ability of OH radicals. (b) PCP-C scavenging ability of DPPH. (c) PCP-C chelating ability of Fe^2+^.

^·^OH radicals can cause tissue damage and cell death and lead to many diseases [39]. Therefore, scavenging OH radicals is one of the important characteristics of the antioxidant defense mechanism. As shown in Figure 3(a), as the concentration of PCP-Cs increased, the ability to remove ^·^OH increased. At the same concentration, the higher the carboxymethylation degree is, the stronger the polysaccharide will be.

#### 3.2.2. DPPH Radical Scavenging Capacity

The DPPH radical has a characteristic absorption peak at 517 nm. When DPPH is reduced to the nonfree radical form DPPH-H by an antioxidant, the purple color of the DPPH radical fades, resulting in a decrease in absorbance [40]. The DPPH radical scavenging ability of PCP-Cs also has a concentration-dependent effect (Figure 3(b)), and PCP-C3 with the highest degree of carboxymethylation has the strongest scavenging ability.

#### 3.2.3. Chelating Effect on Ferrous Ions

Transition metal ions, such as Fe^2+^ and Cu^2+^, can catalyze the chain reaction and generate free radicals, leading to cell oxidative damage [30]. Chelating off metal ions can interrupt chain reaction and prevent oxidative damage. Given that Fe^2+^ has high activity, the ability to chelate this ion is often used to evaluate the antioxidant ability of polysaccharides. As shown in Figure 3(c), when the concentration is 3 g/L, the chelating ability levels of PCP-C0, PCP-C1, PCP-C2, and PCP-C3 to Fe^2+^ are 46.9%, 51.5%, 61.9%, and 63.7%, respectively, and PCP-C3 has the strongest chelating ability.

The three experimental results mentioned above indicate that the carboxymethylation modification of the original PCP-C0 enhances the antioxidant capacity of the polysaccharide.

### 3.3. Characterization of the CaOx Crystal Structure Induced by PCP-Cs

#### 3.3.1. XRD Characterization

**Figure 4:**
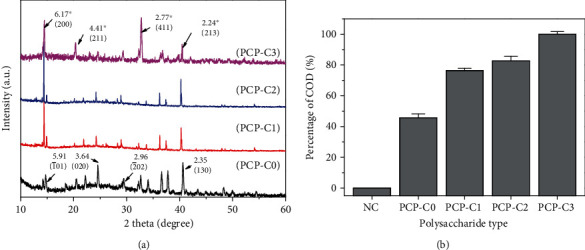
XRD spectrum (a) and COD percentage content (b) of the CaOx crystal formed by PCP-Cs with different COOH contents. (A) PCP-C0. (B) PCP-C1. (C) PCP-C2. (D) PCP-C3. Polysaccharide concentration: 0.4 g/L.

The effects of four kinds of PCP-Cs with different -COOH contents on the formation of CaOx crystals were studied.  Figure 4(a) shows the XRD patterns of CaOx crystals formed in the presence of 0.4 g/L PCP-Cs. The diffraction peaks at crystal plane spacing *d* = 0.593, 0.364, 0.296, and 0.235 nm belong to the $\left(\bar{1}01\right)$, (020), $\left(\bar{2}02\right)$, and (130) crystal planes of calcium oxalate monohydrate (COM), respectively. The peaks at *d* = 0.617, 0.441, 0.277, and 0.224 nm belong to the diffraction peaks of (200), (211), (411), and (213) crystal planes of calcium oxalate dihydrate (COD), respectively [41].

Quantitative calculations according to the *K* value method [12, 26] indicated that as the -COOH content of PCP-Cs increases, the percentage of COD in the crystals induced by PCP-Cs gradually increases (Figure 4(b)). The percentages of COD induced by 0.4 g/L PCP-C0, PCP-C1, PCP-C2, and PCP-C3 were 45.7%, 76.4%, 82.7%, and 100%, respectively.

**Figure 5:**
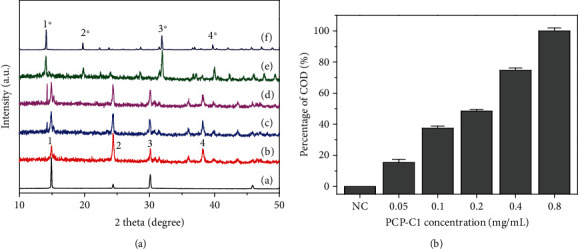
XRD spectrum (a) and COD percentage content (b) of the CaOx crystal formed in the presence of PCP-C1 with different concentrations. (A) 0, (B) 0.05, (C) 0.1, (D) 0.2, (E) 0.4, and (F) 0.8 g/L. The peaks of 1, 2, 3, and 4 belong to ($\bar{1}01$), (020), ($\bar{2}02$), and (130) crystal planes of COM crystals at *d* = 0.593, 0.364, 0.296, and 0.235 nm, respectively. The peaks of 1^∗^, 2^∗^, 3^∗^, and 4^∗^ belong to (200), (211), (411), and (213) crystal faces of COD crystals at *d* = 0.618, 0.442, 0.278, and 0.224 nm, respectively.

With PCP-C1 as a representative, the regulatory effect of polysaccharide concentration on CaOx crystal formation was studied (Figure 5(a)). In the absence of the polysaccharide, only the diffraction peaks of COM appeared. The addition of polysaccharides induced the formation of COD. With increasing PCP-C1 concentration, the intensity of the diffraction peak attributed to COD in the crystal increased continuously, indicating that the percentage of COD induced by the polysaccharide increased. Quantitative calculation also showed that in the presence of 0.05, 0.1, 0.2, 0.4, and 0.8 g/L PCP-C1, the contents of COD in the formed CaOx crystals were 15.4%, 37.4%, 48.4%, 74.7%, and 100%, respectively (Figure 5(b)).

#### 3.3.2. FT-IR Characterization

**Figure 6:**
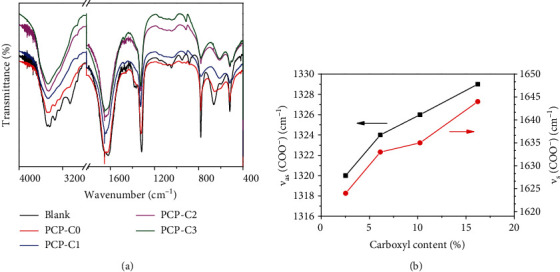
FT-IR spectrum (a) and change of the main absorption peak wavenumber (b) of CaOx crystals generated by regulation in the presence of PCP-Cs with different -COOH contents. *c*(CaOx) = 0.6 mmol/L.

FT-IR detection further supported the regulatory effect of PCP-Cs on CaOx crystal formation (Figure 6(a)). In the absence of the polysaccharide, the following peaks were obtained: carbonyl asymmetric stretching vibration *v*_as_(COO^−^) in the CaOx crystal at 1620 cm^−1^, symmetric stretching vibration *v*_s_(COO^−^) at 1316 cm^−1^, and stretching vibration peaks at 3492-3062 cm^−1^, which belong to the O-H bond of the crystal water. This finding indicated that the calcium oxalate formed is the pure COM crystal [42].

After PCP-Cs were added, *ν*_as_(COO^−^) and *ν*_s_(COO^−^) in the spectrogram undergo a blue shift in different degrees with increasing -COOH content in PCP-Cs (Figure 6(b)). In particular, *ν*_as_(COO^−^) gradually shifts from 1624 cm^−1^ to 1644 cm^−1^ and *ν*_s_(COO^−^) gradually shifts from 1320 cm^−1^ to 1329 cm^−1^, indicating that the COM percentage in the CaOx crystal continuously decreases, while the COD percentage gradually increases. Given that the *ν*_as_(COO^−^) and *ν*_s_(COO^−^) of COD are 1644 and 1329 cm^−1^ [41, 43], respectively, the blue shift values of *ν*_as_(COO^−^) and *ν*_s_(COO^−^) depend on the percentage of COD in the mixture.

In the fingerprint region, the absorption bands of COD crystals were found at 922 and 622 cm^−1^, which differ from those of COM (959, 887, and 667 cm^−1^), of which 887 and 667 cm^−1^ belong to COM C-C stretching vibration and O-C-O in-plane bending vibration, respectively [43].

**Figure 7:**
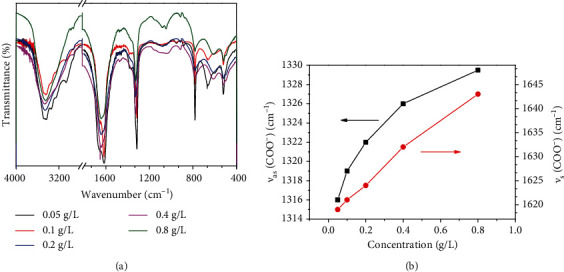
FT-IR spectrum (a) and change of the main absorption peak wavenumber (b) of CaOx crystals generated by regulation in the presence of PCP-C1 with different concentrations. *c*(CaOx) = 0.6 mmol/L.

Figure 7(a) shows the FT-IR spectra of CaOx crystals formed in the presence of PCP-C1 at different concentrations. As the PCP-C1 concentration increased from 0.05 g/L to 0.8 g/L, *ν*_as_(COO^−^) and *ν*_s_(COO^−^) are continuously blue-shifted (Figure 7(b)), where *ν*_as_(COO^−^) increased from 1619 cm^−1^ to 1644 cm^−1^ and *ν*_s_(COO^−^) increased from 1316 cm^−1^ to 1329.5 cm^−1^. The higher the concentration of PCP-C1 is, the higher the percentage of COD in the induced CaOx crystals will be.

### 3.4. Regulation of PCP-Cs on CaOx Morphology

**Figure 8:**
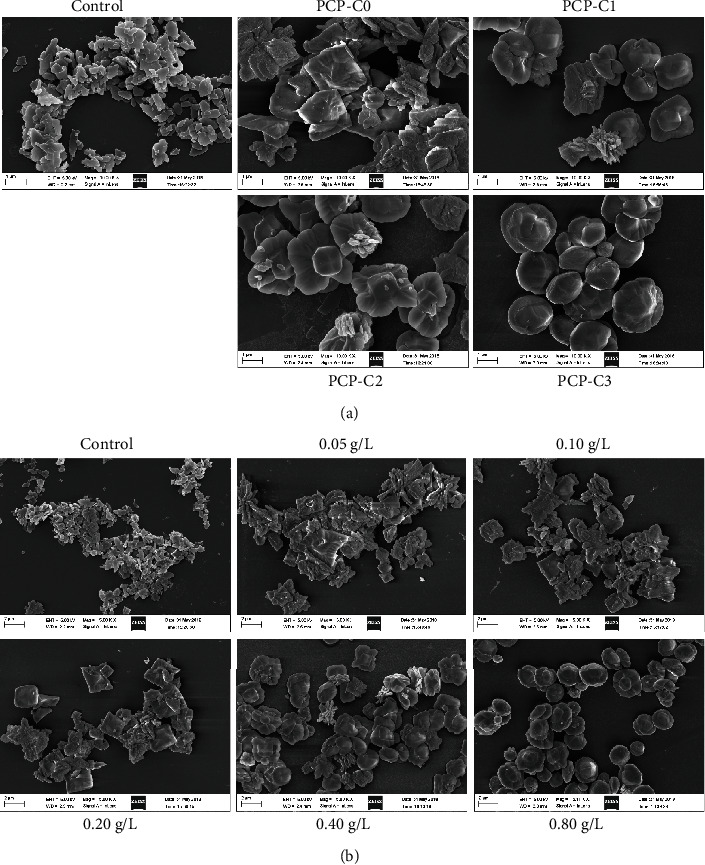
SEM image of the CaOx crystal formed by regulation of PCP-Cs. (a) Effects of -COOH content in PCP-Cs on CaOx morphology. (b) Effects of PCP-C1 concentration on CaOx morphology. (a) (A) PCP-C0, (B) PCP-C1, (C) PCP-C2, and (D) PCP-C3. Polysaccharide concentration: 0.4 g/L. (b) (A) 0, (B) 0.05, (C) 0.1, (D) 0.2, (E) 0.4, and (F) 0.8 g/L.

Figure 8(a) shows the SEM images of CaOx crystals formed in the presence of different PCP-Cs. In the absence of the polysaccharide, most of the crystals formed were COM crystals (Figure 4(a)), which have sharp edges and high aggregation degrees. After adding 0.4 g/L PCP-Cs, COD crystals were formed. With increasing carboxymethylation degrees in PCP-Cs, not only the content of COD increased but also the shape of COD gradually changed from the usual tetragonal bipyramid shape to a round and blunt straw hat shape; meanwhile, the COD induced by PCP-C3 is disc-shaped.

The morphology of CaOx crystals formed by adjusting PCP-C1 concentration is shown in Figure 8(b). With the concentration of PCP-C1 increasing from 0.05 g/L to 0.8 g/L, the proportion of COD in the crystals gradually increased, and the crystal became rounder and blunter, the crystal surface became smoother, and the crystal dispersivity was improved.

### 3.5. Effect of PCP-Cs on the Zeta Potential of Crystals

**Figure 9:**
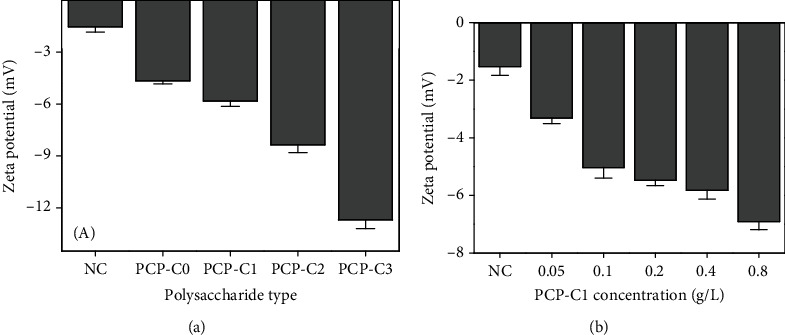
Zeta potential of the formed CaOx crystal regulated by PCP-Cs. (a) Effect of -COOH content of PCP-Cs on the Zeta potential. (b) Effect of PCP-C1 concentration on the Zeta potential.

The particle surface with a high charge density has a large absolute value of the Zeta potential and electrostatic repulsion force between particles, it cannot easily aggregate, and it is stable in solution [43]. The Zeta potentials of CaOx crystals generated by different PCP-Cs are shown in Figure 9(a). With the -COOH content in PCP-Cs increasing from 2.54% to 6.13%, 10.24%, and 16.22%, the Zeta potential decreased from -4.66 mV to -5.82 mV, -8.35 mV, and -12.7 mV; i.e., the higher the -COOH content of polysaccharides is, the larger the absolute value of the Zeta potential on the surface of the CaOx crystal induced. This finding indicated that the more negatively charged PCP-C molecular weight was adsorbed on the crystal surface. For the same polysaccharide PCP-C1, the absolute value of the Zeta potential increased with increasing concentration (Figure 9(b)).

### 3.6. PCP-C Increases Soluble Ca^2+^ Concentration and Decreases CaOx Precipitation

**Figure 10:**
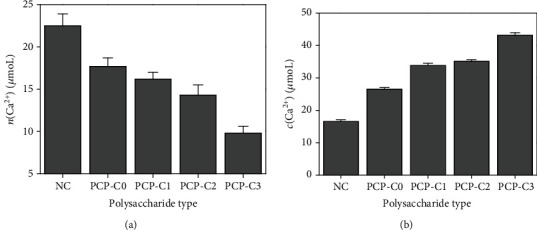
Effect of -COOH content of polysaccharides on the molar amount of CaOx precipitation and concentration of soluble Ca^2+^ in the supernatant. (a) Molar amount of CaOx precipitation. (b) Soluble calcium ion concentration. *c*(PCP‐Cs) = 0.4 g/L.

As shown in Figure 10(a), the molar amount of CaOx precipitates (*n*(CaOx) = 9.8‐17.7 *μ*mol) generated in the presence of different PCP-Cs is lower than that of the blank group (22.5 *μ*mol), and *m*(CaOx) in the presence of PCP-C3 is the least (9.8 *μ*mol). In the presence of different PCP-Cs, the concentration of soluble Ca^2+^ ions (*c*(Ca^2+^) = 26.5 ~ 43.1 *μ*mol/L) in the supernatant is greater than that in the blank group (16.5 *μ*mol/L) (Figure 10(b)). The higher the content of -COOH in the polysaccharide is, the greater the *c*(Ca^2+^) in the supernatant will be.

To verify the reliability of the results in Figure 10, we calculated the sum of the molar amount of soluble Ca^2+^ in the supernatant (*n*(Ca^2+^)) and the molar amount of Ca^2+^ in CaOx precipitates (*n*(Ca^2+^)) (Table 3). The obtained total molar amount of Ca^2+^ ions in each group is 30.7~33.1 *μ*mol, which is consistent with the total molar amount of calcium in the reactant (30.0 *μ*mol).

### 3.7. Thermogravimetric Analysis of CaOx Crystals

**Figure 11:**
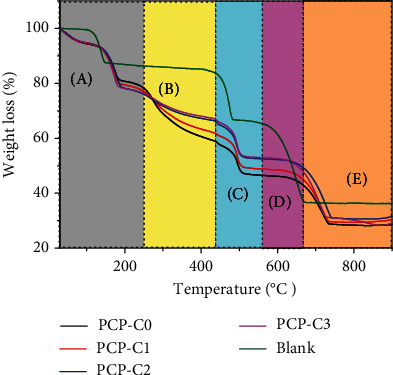
Thermogravimetric analysis curves of CaOx crystals formed in the presence of 0.4 g/L PCP-Cs, respectively.

As shown in Figure 11, the decomposition of CaOx crystals obtained in the blank group without the polysaccharide is divided into three steps, and the weight loss percentages were 12.18% (stage A), 8.33% (stage C), and 28.99% (stage D), consistent with the theoretical weight loss values of 12.33%, 19.17%, and 30.12% of COM (CaC_2_O_4_·H_2_O) decomposed into CaC_2_O_4_, CaCO_3_, and CaO [44].

However, the TGA curve of the CaOx crystal formed after adding 0.4 g/L PCP-C1 differed from that of the blank group due to the formation of COD crystals induced by PCP-C1 and the incorporation of PCP-C1 by the crystal. The CaOx crystals induced by the polysaccharides lose free water and crystalline water at 25°C~128°C (section A). When the temperature continues to rise to 200°C~400°C (section B), the polysaccharide molecules adsorbed on the crystals will undergo thermal decomposition [45]. When the temperature reaches 741°C (section E), the CaOx sample was basically decomposed, and the weight percentages of the final residue of CaOx crystals regulated by PCP-C0, PCP-C1, PCP-C2, and PCP-C3 were 28.72%, 29.60%, 30.95%, and 31.16%, respectively.

The crystals obtained in the blank group without polysaccharides showed no thermogravimetric loss at 200°C~400°C (stage B), while the crystals induced by PCP-Cs underwent thermal decomposition; as such, the weight loss at this stage can be considered to be polysaccharide loss [45], that is, the weight of the polysaccharide incorporated into the crystal. Based on stage B in Figure 11, the proportions of PCP-C0, PCP-C1, PCP-C2, and PCP-C3 incorporated into the crystal were 20.52%, 15.60%, 10.65%, and 9.78%, respectively, and the decomposition temperatures were 205.10°C, 212.00°C, 212.67°C, and 212.05°C, respectively. PCP-C3, which has the highest degree of carboxymethylation, has the lowest proportion of incorporation into the crystals and has a higher decomposition temperature than the original PCP, indicating that PCP-C3 has a stronger specific interaction with the crystals and stronger binding. Given that the incorporation of PCP-Cs makes the decomposition temperature of the “PCP-C crystal” sample higher than that of the pure COM sample, the stability of the “PCP-C crystal” during heating is higher than that of pure crystals without the polysaccharide.

### 3.8. Toxicity Assessment of PCP-Cs on HK-2 Cells

**Figure 12:**
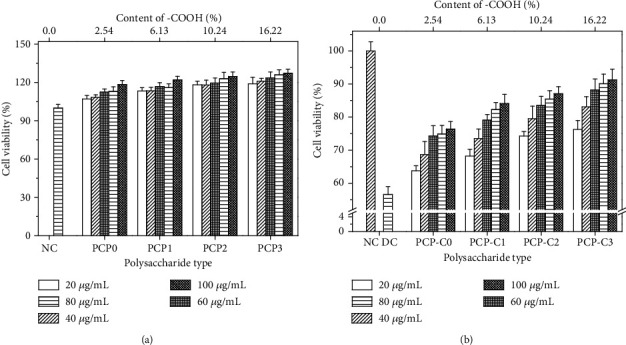
CCK-8 method to detect HK-2 cell viability. (a) Cytotoxicity of PCP-Cs with different concentrations. (b) The viability of HK-2 cells damaged by oxalate with or without PCP-C protection. NC: normal control group; DC: injury control group. Oxalate concentration: 2.8 mM. Injury time: 3.5 h. Protection time: 12 h. Compared with the injury group: ^∗^*P* < 0.05; ^∗∗^*P* < 0.01.

The CCK-8 method was used to detect the toxicity of PCP-Cs with different -COOH contents on HK-2 cells (Figure 12(a)). After HK-2 interacted with PCP-Cs for 24 h, the cell viability was above 100%. Hence, PCP-Cs caused no cytotoxicity on HK-2 cells and promoted cell growth.

### 3.9. PCP-C Protects HK-2 Cells from Damage

Figure 12(b) shows the changes in HK-2 cell viability before and after preprotection with different carboxymethylated PCP-Cs. After 2.8 mM oxalate oxidation injury, the cell activity decreased from 100% of the control group to 56.6%. However, with different concentrations of PCP-C preprotection, the cell viability of the injured group was significantly higher than that of the injured group.

For the same polysaccharide with concentration < 100 *μ*g/L, the preprotection of PCP-Cs shows a concentration effect; i.e., the higher the concentration of PCP-Cs is, the better the protection effect will be.

At the same concentration, the cell viability of different polysaccharide protection groups followed the order of PCP-C0<PCP-C1<PCP-C2<PCP-C3; i.e., PCP-C3 with the highest -COOH content had the best protection effect. At 100 *μ*g/L, the cell activity under PCP-C3 protection reached 90.3%.

### 3.10. PCP-C Protection Reduces Reactive Oxygen Species (ROS) Production Caused by Oxalate

**Figure 13:**
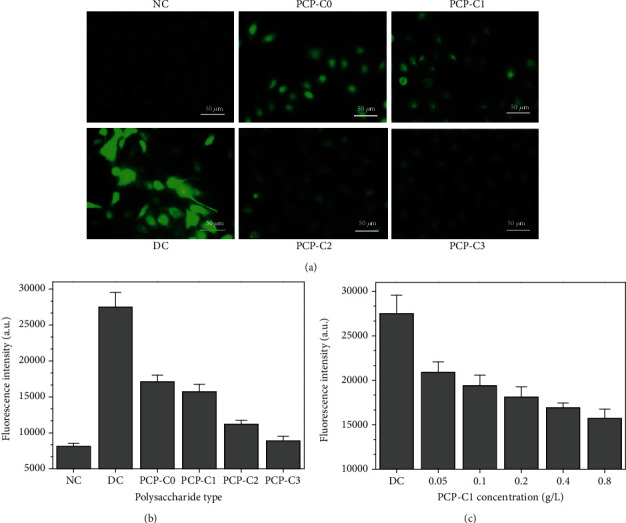
Effects of PCP-Cs before and after protection on ROS expression in HK-2 cells. (a, b) Effects of COOH content in PCP-Cs. Polysaccharide concentration: 100 *μ*g/mL. (c) Effect of PCP-C1 concentration. NC: normal control group; DC: injury control group. Oxalate concentration: 2.8 mM. Injury time: 3.5 h. Protection time: 12 h.

Figure 13(a) shows the ROS level changes in HK-2 cells before and after PCP-C preprotection. In the normal group, the cells grew tightly, with the lowest ROS fluorescence intensity, that is, less ROS. The intensity of ROS fluorescence in cells damaged by oxalate was significantly enhanced. After 100 *μ*g/mL of four PCP-Cs was preprotected, the fluorescence intensity of ROS in cells became weak, which was between the normal group and the protection group (Figure 13(b)), indicating that PCP-Cs can resist oxidative damage from oxalate after protection.

At the same time, the preprotection effect of PCP-C1 with different concentrations on HK-2 cells was also detected by a microplate reader (Figure 13(c)). With the increase of PCP-C1 concentration, the ROS level of cells gradually decreased, indicating that PCP-C1 has a concentration effect when protecting cells.

### 3.11. PCP-C Protection Increases Antioxidant Ability and Reduces Oxidative Damage

**Figure 14:**
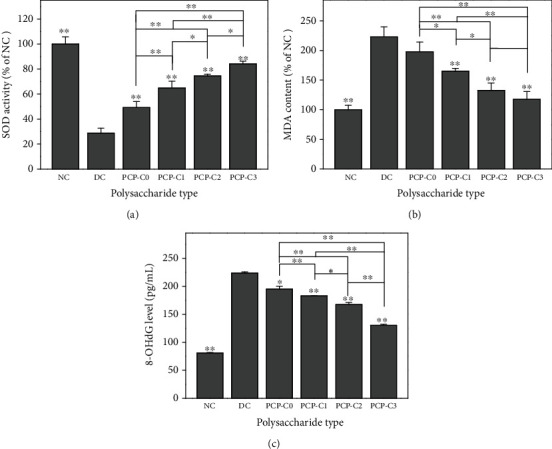
Effects of PCP-Cs before and after protection on SOD activity (a), MDA content (b), and 8-OHdG expression (c) in HK-2 cells. NC: normal control group; DC: injury control group. Oxalate concentration: 2.8 mM. Injury time: 3.5 h. Protection time: 12 h. Compared with the injury group: ^∗^*P* < 0.05; ^∗∗^*P* < 0.01.

The reduction of SOD activity in the organism implies decreased ability to resist free radical-induced damage in the organism. The SOD activity in the oxalate-damaged group was decreased to 28.78 ± 4.02% of the control value. After the protection by PCP-C0, PCP-C1, PCP-C2, and PCP-C3, the SOD activity increased to 49.22%, 64.78%, 74.57%, and 84.18%, respectively (Figure 14(a)).

The change of MDA content usually reveals the level of lipid peroxidation in vivo and indirectly reflects the degree of cell injury. After oxalate was used to damage the cells, the MDA content increased to 222.84% of the control group. The MDA content was obviously reduced in the PCP-C-protected groups; the released content was reduced to 197.97% (PCP-C0), 164.84% (PCP-C1), 132.68% (PCP-C2), and 117.65% (PCP-C3) of the control group (Figure 14(b)). PCP-C3 with the highest -COOH contents has the strongest ability to inhibit MDA release.

The concentration of 8-OHdG is considered a marker of oxidative DNA damage. The 8-OHdG expression was low in the normal control cells (80.65 pg/mL). In the oxalate-damaged group, the 8-OHdG expression obviously increased to 223.61 pg/mL. The expression level of 8-OHdG in the PCP-C-protected groups obviously decreased compared to the oxalate-damaged group. The 8-OHdG concentration decreased to 195.31–130.25 pg/mL (Figure 14(c)). PCP-C3 with the highest -COOH contents presented the optimum DNA protection ability.

## 4. Discussion

### 4.1. Carboxymethylation Modification of *P*. *cocos* Polysaccharide

#### 4.1.1. Influence of Reaction Conditions

Carboxymethylation of PCP-C0 includes two steps. Firstly, NaOH reacts with -OH of polysaccharide molecules to generate alkoxide groups on the PCP-C0; then, the carboxymethyl group was formed in polysaccharide alkoxide and monochloroacetic acid (ClCH_2_COOH) by SN2 reaction [14, 15]. The factors that affect the degree of substitution of polysaccharide carboxymethylation include reactant concentration, temperature, and reaction time (Table 4). In this experiment, three carboxymethylated polysaccharides PCP-C1, PCP-C2, and PCP-C3 were obtained at 60°C by changing the reaction time and reactant concentration. The contents of -COOH were 6.13%, 10.24%, and 16.22%, respectively, which were greater than 2.54% of the initial polysaccharide PCP-C0. Duan et al. [33] carboxymethylated the natural polysaccharide RNP extracted from *blackcurrant* fruits. As the temperature increased from 50°C to 70°C, the substitution degree increased from 0.57 to 1.10, which may be due to the increased temperature enhancing the solubility of polysaccharide alkoxide, resulting in better contact between polysaccharide alkoxide and ClCH_2_COOH. When the temperature increased further, the substitution degree decreased, which is attributed to the fact that too high a temperature is conducive to the occurrence of side reactions. Liu et al. [46] modified *Sarcandra glabra* polysaccharide by carboxymethylation and found that with the extension of reaction time, the degree of carboxymethylation first increased and then decreased. Prolonging the reaction time helps polysaccharides to expand and promote ClCH_2_COOH to enter the polysaccharide molecules. However, too long reaction time at higher temperatures is conducive to the formation of side reaction products, such as glycolate.

#### 4.1.2. Structural Identification of Carboxymethylated Polysaccharides

From ^1^H NMR and ^13^C NMR spectra (Figure 2), PCP-C0 was composed of glucose, galactose, and fucose, and the main polysaccharide residues were *α*-D-Gal, *α*-(1-6)-L-Fuc, *α*-(1-3)-L-Fuc, *β*-(1-3)-D-Glc, and *β*-(1-3)-D-Gal, which was basically consistent with the structure reported by Meikuang et al. [47] and Wang et al. [19]. After carboxymethylation, the ^13^C NMR of PCP-C1 showed a new absorption peak at 177.8 ppm, which showed the characteristic absorption peak of the carboxymethyl group (-CH_2_COOH) [23, 48].

FT-IR spectra show that the main chain structures of the four carboxymethylated polysaccharides are similar, but the carboxymethylated polysaccharides have a new characteristic absorption peak at 1326.5 cm^−1^, which is attributed to -CH_2_COOH groups. The experimental results are consistent with the ^13^C NMR results.

#### 4.1.3. Enhancement of Antioxidant Activity of Polysaccharides after Carboxymethylation

Carboxymethylation modification can enhance the antioxidant activity of polysaccharide, which is related to the following factors:

1. The introduction of the substituent group -CH_2_COOH changes the configuration of polysaccharide, which weakens the dissociation energy of hydrogen bonds in polysaccharide molecules [49] and improves the hydrogen supply capacity of polysaccharide
2. The biological activity of polysaccharides is positively correlated with their water solubility. Carboxymethylation modification can increase the water solubility of polysaccharides [50], so the degree of freedom of polysaccharides is increased, and the ability to exert activity is stronger
3. Polysaccharides have two mechanisms for antioxidant activity. One is that polysaccharides inhibit the generation of free radicals by chelating transition metal ions (such as Fe^2+^ and Cu^2+^ ions) [51, 52]. The other is that polysaccharides can provide single electrons or hydrogen atoms for free radicals, thus terminating free radical chain reaction and achieving the purpose of scavenging free radicals. As the content of -COOH in carboxymethylated polysaccharide increases significantly, its ability to chelate transition metal ions and provide single electron or hydrogen atoms all increases, thereby increasing the antioxidant activity

Shi et al. [34] extracted the original crude *Enteromorpha prolifera* polysaccharide (PE) with a molecular weight of 1400 kDa, degraded it to obtain a low molecular weight polysaccharide DPE with a molecular weight of 44 kDa, and carboxymethylated the DPE to obtain CDPE. The DPPH scavenging ability, hydroxyl radical scavenging ability, and reducing ability of the three polysaccharides are CDPE>DPE>PE. Li et al. [53] also showed that the radical scavenging ability and total antioxidant ability of the carboxymethylated degraded *Sargassum fusiforme* polysaccharide (CDPSSF) are significantly higher than those of the degraded polysaccharide (DPSF); the antioxidant activity of DPSF is higher than that of the nondegraded polysaccharide (PSF).

### 4.2. Carboxymethylated Polysaccharides Have Stronger Ability to Regulate CaOx Crystal Growth

**Figure 15:**
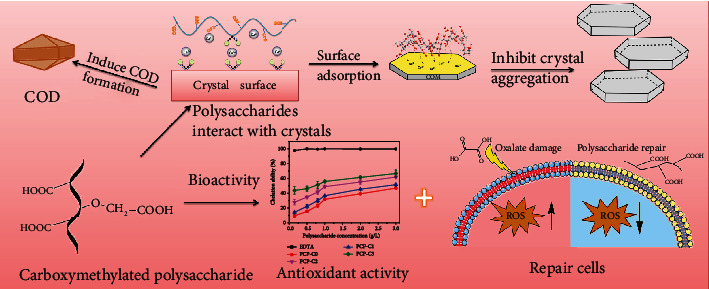
Model diagram of biological activity of carboxymethylated polysaccharides (PCP-Cs) and their regulatory effect on CaOx crystals.

Carboxymethylated polysaccharides can significantly inhibit COM crystal growth, induce COD crystal formation, and inhibit crystal aggregation (Figure 15).

First, after carboxymethylation, the content of -COOH in polysaccharides increases, which can complex a large number of Ca^2+^ ions in the system to inhibit the combination of Ca^2+^ and Ox^2-^ to form CaOx precipitates. The higher the degree of carboxymethylation is, the stronger the inhibitory ability will be.

Second, Ca^2+^ ions on the polysaccharide surface are highly enriched, forming a high energy interface because the carboxymethylated polysaccharide has enhanced ability to complex Ca^2+^ ions [54]. After Ca^2+^ ions are adsorbed, their degree of freedom decreases and the energy state of calcium increases, which are conducive to promoting the formation of COD [26].

Third, the results of thermogravimetric analysis (Figure 11) showed that during the process of PCP-Cs regulating the growth of CaOx, PCP-Cs were adsorbed or incorporated into the CaOx crystals (Table 5). The adsorption of PCP-Cs resulted in the accumulation of high-density negative charges on the surface of the CaOx crystals, causing the absolute value of the Zeta potential to increase (Figure 9), thereby inhibiting the aggregation of CaOx crystals. Zhang et al. [6] showed that *Sargassum* polysaccharide (SGP) can significantly inhibit the aggregation of COM crystals; the inhibition rate of SGP at a concentration of 0.5 g/L on the aggregation of COM crystals is as high as 76.8%.

Fourth, the Ca^2+^ ions at the tip and edge of the crystal are easy to coordinate with -COOH of polysaccharides in solution due to the dissociation-precipitation equilibrium between a large number of -COOH groups in carboxymethylated polysaccharide and the formed CaOx crystals. The continuous dissociation-precipitation finally makes the CaOx crystal blunt (Figure 8). Given that the damage degree of the sharp-edged COM crystal to renal epithelial cells is higher than that of the blunt COD crystal and the affinity between the COM with the positively charged surface and the damaged renal epithelial cells with the negatively charged surface is higher than that of the COD crystal [53, 54], COD is easier to be excreted out of the body with urine. PCP-Cs induce the formation of more or even all COD crystals, thereby reducing the risk of CaOx kidney stone formation.

As shown in Figure 8, the crystal size of the control group is significantly smaller due to the maximum supersaturation of CaOx in the control system without the polysaccharide and the maximum nucleation rate of the crystal. Under the condition of fixing the total amount of CaOx, rapid nucleation reduced the average size of each crystal. This finding also explains why PCP-C3 has the strongest complexing ability with CaOx, but the crystal size induced by PCP-C3 does not decrease; i.e., the number of formed crystals is small, but the size is still large.

### 4.3. Carboxymethylated Polysaccharide Has Stronger Ability to Protect Renal Epithelial Cells from Oxalate Toxicity

High-level ROS can react with intracellular macromolecules rapidly, impairing the function of normal cells and even leading to cell death [51]. The increase of SOD activity in the organism implies an elevated ability to resist free radical-induced damage in the organism. The change of MDA content reveals the level of lipid peroxidation and indirectly reflects the degree of cell injury. The concentration of 8-OHdG is considered a marker of oxidative DNA damage. After protecting HK-2 cells with different degrees of carboxymethylated PCP-Cs, oxidative damage from oxalate can be alleviated, thereby increasing cell viability (Figure 12(b)) and superoxide dismutase activity (Figure 14(a)) and decreasing ROS level (Figure 13), MDA content (Figure 14(b)), and 8-OHdG expression (Figure 14(c)). Thus, PCP-Cs can protect HK-2 cells from oxidative damage by oxalate and improve cellular antioxidant ability. PCP-C with a higher carboxymethylation degree has a stronger protection ability.

Zhang et al. [55] used thrombin to induce inflammation of rat endothelial progenitor cells (EPC) and found that *Astragalus* polysaccharide (APS) has a protective effect on injured endothelial progenitor cells. APS can block the nuclear factor kappa B (NF-*κ*B) signaling in EPC, upregulate the expression of the vascular endothelial growth factor (VEGF) and its receptor, and inhibit the expression of intercellular adhesion molecule-1 (ICAM-1) induced by thrombin, thereby protecting cells from damage. Li et al. [56] found that Chinese chive polysaccharide (CCP) can inhibit the oxidative damage to the kidney of mice with chronic renal failure (CRF). Sulfated polysaccharides from *Codium fragile* polysaccharide (CFCE-PS) have protective effects on H_2_O_2_-induced oxidative stress-damaged cells [57]. After being protected by CFCE-PS, the cell vitality is enhanced, the intracellular ROS level is reduced, and the cell apoptosis is inhibited.

Our results indicated that carboxymethylated PCP-Cs have the ability to inhibit calcium oxalate formation and resist the oxidative damage of oxalate in vitro. However, more in-depth molecular mechanisms and verification in vivo need to be further studied in the future.

## 5. Conclusions

Carboxymethylation of PCP-C0 with -COOH content of 2.54% was carried out, and three carboxymethylated polysaccharides with -COOH contents of 6.13%, 10.24%, and 16.22% were obtained. Compared with PCP-C0, the carboxymethylated polysaccharides (PCP-Cs) can significantly enhance its antioxidant capacity, protect renal epithelial cells from oxidative damage, inhibit COM growth, induce COD formation, inhibit crystal aggregation, and increase the concentration of soluble Ca^2+^ ions in the system, all of which are beneficial to inhibit the formation of CaOx kidney stones. With increasing carboxymethylation degrees in PCP-Cs, its biological activity gradually increases. This study suggests that carboxymethylation of polysaccharides is an effective method to enhance biological activity and anticalculus ability. The higher the degree of carboxymethylation is, the stronger the activity of polysaccharides will be.

## Figures and Tables

**Table 1:** Chemical shifts corresponding to the ^1^H NMR spectrum of carboxymethylated polysaccharide PCP-C0.

Polysaccharide type	Sugar residue	^1^H chemical shift (ppm)					
		H-1	H-2	H-3	H-4	H-5	H-6
PCP-C0	*β*-(1-3)-D-Glc	4.71	—	—	—	—	—
	*β*-(1-3)-D-Gal	4.59	—	—	—	—	—
	*α*-D-Gal	5.14	3.98	3.67	3.58	3.81	3.66
	*α*-(1-6)-L-Fuc	4.95	3.59	3.98	3.89	4.08	1.14
	*α*-(1-3)-L-Fuc	4.95	3.88	3.75	3.89	4.08	1.14

**Table 2:** Chemical shifts corresponding to ^13^C NMR spectra of carboxymethylated polysaccharides (PCP-Cs).

Polysaccharide type	Sugar residue	^13^C chemical shift (ppm)							
		C-1	C-2	C-3	C-4	C-5	C-6	-CH_3_	C-7
PCP-C0	*β*-(1-3)-D-Glc	102.53	74.12	95.89	69.58	75.74	60.75	—	—
	*β*-(1-3)-D-Gal	103.7	73.5	86.9	69.1	77.0	61.6	—	—
	*α*-D-Gal	—	—	73.6	67.0	70.5	61.3	—	—
	*α*-(1-6)-L-Fuc	98.2	67.7	70.5	71.6	67.3	—	15.8	—
	*α*-(1-3)-L-Fuc	98.2	67.0	69.7	71.6	67.3	—	15.8	—
PCP-C1		102.8	73.1	79.2	69.6	74.8	60.9	—	177.8

**Table 3:** Effect of PCP-Cs on the CaOx crystal phase, soluble Ca^2+^ ion concentration in solution, and CaOx precipitation mass.

Polysaccharide	Carboxyl content (%)	COD percentage (%)	*c*(Ca^2+^) (*μ*mol/L)	*n*(Ca^2+^) (*μ*mol)	Mass of CaOx (g)	*m*(CaOx)^∗^ (*μ*mol)	Total Ca^2+^ (*μ*mol)
Blank	—	0	16.5	8.2	0.00328	22.5	30.7
PCP-C0	2.54	45.7	26.5	13.3	0.00274	17.7	30.9
PCP-C1	6.13	76.4	33.8	16.9	0.00256	16.2	33.1
PCP-C2	10.24	82.7	35.1	17.5	0.00226	14.3	31.9
PCP-C3	16.22	100	43.1	21.6	0.00160	9.8	31.4

^∗^When calculating the precipitation mass of CaOx *m*(CaOx), the crystal waters in COM and COD should be considered; that is, the molar mass of COM and COD is 146 and 164 g/mol, respectively.

**Table 4:** Carboxymethylation conditions of PCP-Cs and carboxyl content of polysaccharides.

Polysaccharide type	Isopropanol volume (mL)	NaOH volume (mL)	Chloroacetic acid amount (g)	Reaction time (h)	Temperature	COOH content (%)
PCP-C0	—	—	—	—		2.54
PCP-C1	15	10	2.63	2	60	6.13
PCP-C2	15	10	2.63	8	60	10.24
PCP-C3	10	15	6.00	4	60	16.22

**Table 5:** TGA curve analysis of CaOx crystals formed in the presence of 0.4 g/L PCP-Cs and the blank group, respectively.

PCP-Cs	A		B		C		D		Residual weight (%)
	Decomp. *T* (°C)∗^1^	Weight loss (%)	Decomp. *T* (°C)	Weight loss (%)∗^2^	Decomp. *T* (°C)	Weight loss (%)	Decomp. *T* (°C)	Weight loss (%)	
Blank	95.73	12.08	—	—	409.14	18.33	552.94	28.99	36.55
PCP-C0	110.33	13.49	205.10	20.52	427.84	12.61	602.29	17.36	28.72
PCP-C1	106.94	14.89	212.00	15.60	427.14	13.17	598.17	18.85	29.60
PCP-C2	107.68	16.62	212.67	10.65	426.54	13.93	605.64	21.23	30.95
PCP-C3	102.97	16.87	212.05	9.78	424.50	14.24	589.75	21.57	31.16

^∗^^1^Decomp. *T*: decomposition temperature. ^∗^^2^The weight loss in stage B is the content of polysaccharides.

## Data Availability

All the data supporting the results were shown in the paper and can be available from the corresponding authors.
